# Chronic atrophic gastritis and risk of incident upper gastrointestinal cancers: a systematic review and meta-analysis

**DOI:** 10.1186/s12967-023-04736-w

**Published:** 2024-05-06

**Authors:** Junqiu Li, Jielu Pan, Dinghong Xiao, Nan Shen, Ruiqing Wang, Hongyv Miao, Peimin Pu, Haiyan Zhang, Xiao Yv, Lianjun Xing

**Affiliations:** Department II of Digestive Diseases, Longhua Hospital Affiliated to Shanghai University of Traditional Chinese Medicine, Shanghai, 200032 China

**Keywords:** Chronic atrophic gastritis, Upper gastrointestinal cancers, Gastric cancer, Oesophageal cancer, Oesophagogastric junction cancer, Meta-analysis

## Abstract

**Background:**

Previous literature has explored the relationship between chronic atrophic gastritis (CAG) and isolated cancers within the upper gastrointestinal cancers; However, an integrative synthesis across the totality of upper gastrointestinal cancers was conspicuously absent. The research objective was to assess the relationship between CAG and the risk of incident upper gastrointestinal cancers, specifically including gastric cancer, oesophageal cancer, and oesophagogastric junction cancer.

**Methods:**

Rigorous systematic searches were conducted across three major databases, namely PubMed, Embase and Web of Science, encompassing the timeline from database inception until August 10, 2023. We extracted the necessary odds ratio (OR) and their corresponding 95% confidence interval (CI) for subsequent meta-analysis. Statistical analyses were conducted using Stata 17.0 software.

**Results:**

This meta-analysis included a total of 23 articles encompassing 5858 patients diagnosed with upper gastrointestinal cancers. CAG resulted in a statistically significant 4.12-fold elevated risk of incident gastric cancer (OR = 4.12, 95% CI 3.20–5.30). Likewise, CAG was linked to a 2.08-fold increased risk of incident oesophageal cancer (OR = 2.08, 95%CI 1.60–2.72). Intriguingly, a specific correlation was found between CAG and the risk of incident oesophageal squamous cell carcinoma (OR = 2.29, 95%CI 1.77–2.95), while no significant association was detected for oesophageal adenocarcinoma (OR = 0.62, 95%CI 0.17–2.26). Moreover, CAG was correlated with a 2.77-fold heightened risk of oesophagogastric junction cancer (OR = 2.77, 95%CI 2.21–3.46). Notably, for the same type of upper gastrointestinal cancer, it was observed that diagnosing CAG through histological methods was linked to a 33–77% higher risk of developing cancer compared to diagnosing CAG through serological methods.

**Conclusion:**

This meta-analysis indicated a two- to fourfold increased risk of gastric cancer, oesophageal cancer, and oesophagogastric junction cancer in patients with CAG. Importantly, for the same upper gastrointestinal cancer, the risk of incident cancer was higher when CAG was diagnosed histologically compared to serological diagnosis. Further rigorous study designs are required to explore the impact of CAG diagnosed through both diagnostic methods on the risk of upper gastrointestinal cancers.

**Supplementary Information:**

The online version contains supplementary material available at 10.1186/s12967-023-04736-w.

## Introduction

Within the global healthcare arena, cancer plays a dual role, being both a disease of significant global interest and a principal factor in clinical mortality. It is characterized by a protracted disease course, a predisposition for deterioration, low survival rates, and a significant economic burden. With the ageing of the population and an increase in cancer risk factors, the incidence and mortality of cancer have also risen.

Upper gastrointestinal cancers, comprising gastric cancer (GC), oesophagogastric junction cancer (OJC), and oesophageal cancer (OC); Oesophageal cancer is mainly classified into two subtypes: oesophageal adenocarcinoma (OAC) and oesophageal squamous cell carcinoma (OSCC). In 2019, there were approximately 23.6 million new cancer cases reported worldwide, with upper gastrointestinal cancers representing about 7.6% of these cases; Meanwhile, the worldwide cancer-related mortality rate reached an estimated 10.0 million, and upper gastrointestinal cancers were responsible for roughly 14.6% of these deaths [1]. The etiopathogenesis and progression of upper gastrointestinal cancers are closely linked to numerous factors, including diet, lifestyle, and others [2, 3]. Notably, Chronic atrophic gastritis (CAG) has captured the attention of researchers as a potential risk factor. This association is consistent with the involvement of chronic inflammation in cancer development [4, 5].

CAG is a chronic inflammatory disease characterized by the reduction or loss of gastric mucosal glands, with or without metaplasia of the intestinal epithelium or pyloric glands. A primary etiological factor in the development of this disease is the infection of H. pylori [6–8]. Upon infection, the gastric mucosa undergoes an intense inflammatory response, resulting in tissue damage and an increased risk of cancer [9]. Subsequently, some researchers initiated studies of the associations between CAG and upper gastrointestinal cancers. Over the last 15 years, the majority of studies have primarily centred around meta-analyses examining the relationship between CAG and GC [10, 11]. However, there has been relatively limited research concerning the relationship between CAG and OC or OJC. Notably, it was not until 2010 that a meta-analysis was published, reporting on the risk of gastric atrophy in the development of OAC and OSCC [12]. At present, no exhaustive meta-analysis offers a comprehensive assessment of the risk of upper gastrointestinal cancers in relation to CAG. Meanwhile, with advances in medical science and technology, the primary diagnostic modality for CAG has shifted towards histological methods, whereas previous studies mainly used serologic diagnostic modalities. However, Whether the risk relationship between CAG diagnosed using these two diagnostic methods and upper gastrointestinal cancers is consistent remains unclear and has not been clearly reported.

Consequently, we conducted this systematic review and meta-analysis to comprehensively and accurately assess the magnitude and nature of the relationship between CAG and the incidence risk of upper gastrointestinal cancers. Furthermore, we aimed to report the extent of risk associated with the diagnosis of CAG through histological and serological methods for the development of upper gastrointestinal cancers.

## Materials and methods

This study was conducted in accordance with the recommendations of the Preferred Reporting Items for Systematic Reviews and Meta-Analyses (PRISMA) [13] and was registered with the PROSPERO (CRD42023455940).

### Search strategy

We systematically searched databases (PubMed, Embase, Web of Science) using a combination of search terms and free phrases to assess the risk association between CAG and upper gastrointestinal cancers. The search included articles published from the creation of the database through August 10, 2023. The search strategies used for each database are displayed in Additional file 1: File 1 Search strategy.

### Inclusion and exclusion criteria

The inclusion criteria were as follows: (1) Case–control studies, nested case–control studies, or cohort studies; (2) To investigate the risk relationship between CAG and upper gastrointestinal cancers; (3) The diagnosis of CAG is based on endoscopic histology or serological methods. (4) The study involved human participants, with no restrictions on race or gender, and all individuals were aged 18 years or older. (5) The main outcome was the incidence risk of upper gastrointestinal cancers, which was measured using odds ratio (OR).

The exclusion criteria were as follows: (1) Case reports, reviews, commentaries, animal and cell studies, as well as cross-sectional research; (2) Duplicate publications; (3) Literature with missing research data and inability to extract the required data; (4) Non-English literature; (5) Newcastle–Ottawa Scale score (NOS) < 7.

### Data extraction and quality assessment

According to the inclusion and exclusion criteria, two researchers (JQL and XY) independently screened titles and abstracts that met the requirements. Subsequently, they obtained and read the full texts, selecting articles that met the specified criteria. According to the data extraction guidelines for systematic reviews and meta-analyses [14], two researchers independently extracted the following information: study design, study’s author and year of publication, country, sample size, outcomes, study period, sex, diagnosis of CAG, assessment of cancer, adjustment for covariates, participant source and NOS score.

If a study did not clearly give a standard definition of gastric atrophy, we defined it as atrophic gastritis based on histological evidence of gastric mucosal atrophy and intestinal metaplasia. This was based on an expert review of atrophic gastritis updated by the American Gastrointestinal Association [15]. Hence, when independently assessing the risks associated with gastric mucosal atrophy and intestinal metaplasia in the literature, we regarded them as separate studies. Similarly, when conducting separate risk assessments for histology and serology, we also treated them as separate studies. If the literature independently assessed the risks of non-cardia cancer and cardia cancer, we extracted relevant data on non-cardia cancer for the study of gastric cancer incidence risk. In accordance with the classification of oesophagogastric junction adenocarcinoma [16], we included cardia cancer-related data in the study of oesophagogastric junction cancer incidence risk.

We undertook a qualitative evaluation of the included literature utilizing the Newcastle–Ottawa Scale (NOS). This assessment was carried out independently by two researchers (JQL and JLP). The NOS scale comprises three aspects of evaluation, with scores ranging from 0 to 9. In this study, the quality assessment scores for all screened literature were 7 or higher. Therefore, the literature screened in this study was considered to be of high quality [17].

Any disagreements encountered during the processes of data extraction and quality assessment were addressed through discussions with the senior author (LJX).

### Statistical methods

The meta-analysis was conducted by comparing the risk of upper gastrointestinal cancers between patients with and without CAG. We extracted OR, hazard ratio (HR), and relative risk (RR) from the eligible literature. Given the relatively low risk of upper gastrointestinal cancers, during the data analysis, the extracted HR and RR were approximated to be equal to the OR [18]. We used the OR and its corresponding 95% confidence interval (CI) for statistical analysis.

The statistical analyses were performed using Stata 17.0 software. To assess heterogeneity, we used the Q-test and the I^2^ value. When I^2^ > 50% or P < 0.1, we considered significant heterogeneity among the studies, allowing for the adoption of a random-effects model. Otherwise, a fixed-effects model was used; Additionally, in order to explore the sources of heterogeneity, we conducted subgroup analyses based on the diagnosis of CAG, study type, participant source, region, year of publication, and NOS score. Sensitivity analysis was conducted to evaluate the robustness and reliability of the results. Funnel plots and Egger's test were used to analyze publication bias.

## Results

### Search results

Initially, we retrieved a total of 16,039 articles, which included 3422 from PubMed, 5489 from Embase, and 7128 from Web of Science. Among these, 6691 duplicates were identified and subsequently excluded, followed by the exclusion of 9,283 irrelevant articles after a screening of titles and abstracts. We conducted a comprehensive search of the full text of 65 articles, excluding one article that was unavailable. Following a detailed examination of the full texts, we excluded 41 studies for various reasons, including 21 studies lacking relevant outcomes, 11 studies with unrelated outcomes, 2 studies were letters, 1 study was review, 1 study in a non-English language, 1 study was conference abstract, and 4 studies with a NOS < 7.

**Fig. 1:**
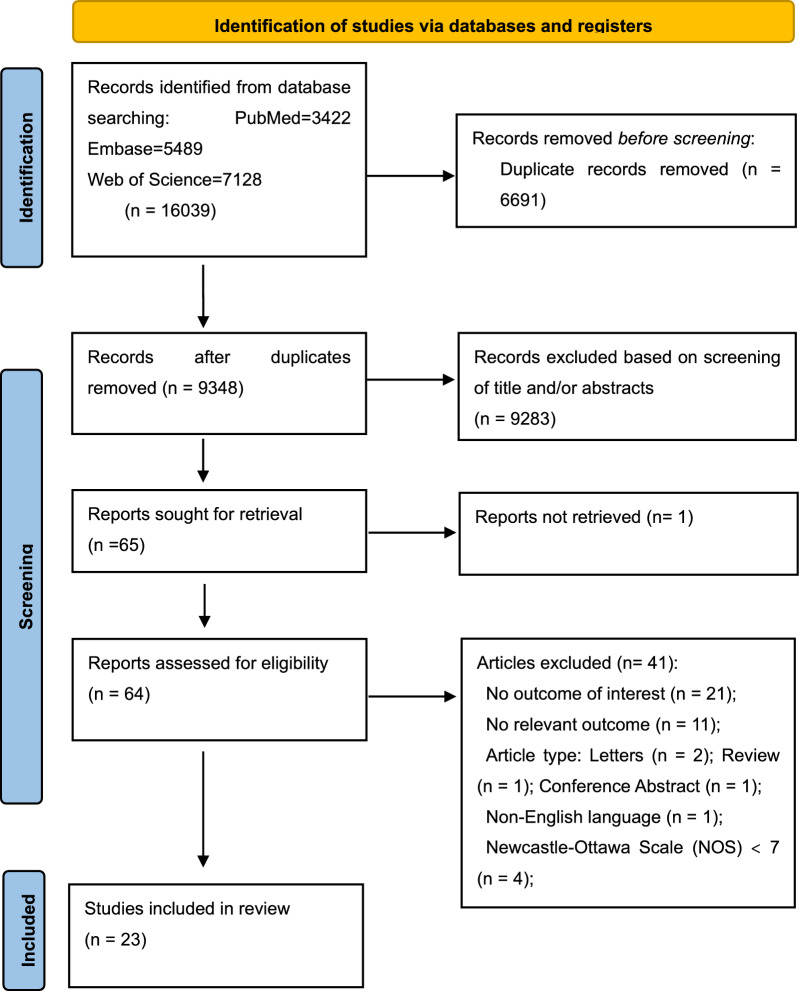
Flow chart of literature screening

Finally, a total of 23 articles involving 5858 patients diagnosed with upper gastrointestinal cancers were incorporated into this study. The flowchart of the study screening is shown in Fig. 1 (Page 30).

### Characteristics of included studies

We have identified 23 articles, containing 36 studies, to assess the relationship between CAG and the risk of incident upper gastrointestinal cancers [19–41]. Among these, 13 studies analyzed gastric cancer, 15 studies examined oesophageal cancer, and 8 studies explored oesophagogastric junction cancer. CAG was diagnosed using endoscopic histological methods (found in a total of 7 articles) and serological methods (found in a total of 18 articles), with 2 articles conducting research on both of these diagnostic approaches.

In the included literature, there were 10 case–control studies, 6 nested case–control studies, and 7 cohort studies. Detailed characteristics of these incorporated studies can be found in Table 1 (Pages 31–35). These studies were published between 1995 to 2023 and collectively involved 5858 patients diagnosed with upper gastrointestinal cancers, with two articles exclusively focused on male populations. For the source of study participants, there were 5 articles based on hospital-based research, 17 articles based on population-based research, and 1 article based on a combination of population and clinic-based research. In terms of the regional distribution of the study, 10 articles were performed in Europe, 11 articles in Asia (including 8 articles in Japan), and 2 articles in the Americas. According to the NOS scale score, 6 studies received a score of 9, 18 studies achieved a score of 8, and 12 studies were rated with a score of 7.

**Table1:** Baseline characteristics of included studies

Study design	Refs.	Country	Sample size	Outcomes	Study period (year)	Sex	Diagnosis of CAG	Assessment of cancer	Adjustment for covariates	Participant source	NOS
Case–control	Akiyama et al. [26]	Japan	Case:253; control:253	OC (OSCC)	1997–2008	M/F	Histology: gastric mucosal atrophy open-type 2 and 3	Complete medical record	Age, sex, BMI, current regular drinking and smoking habits	H-B	8
	Almodova et al. [27]	Latin-America	Case:49; control:49	OC (OSCC)	2011–2012	M/F	Histology: mucosal atrophy, intestinal metaplasia, and dysplasia	Endoscopy aspects and final pathology report from the hospital	Age, sex	H-B	7
	Anderson et al. [28]	Ireland	Case:122; control:240	OC (OAC)	2002–2005	M/F	Pepsinogen I/II ratio < 3 and < 7	Clinical and histological records, data and samples from FINBAR study	Age, gender, measures of socioeconomic status, body mass index, smoking and alcohol consumption, location, parental occupation and household density during childhood	P-B, C-B	7
			case:80; control:240	OJC			Pepsinogen I/II ratio < 3 and < 7				7
	Ekheden et al. [32]	China	case:1120; control:1911	OC (OSCC)	2010–2014	M/F	Pepsinogen I < 55 μg/L	Hospitals and cancer registries	Age, sex, education, marital status, occupation, family wealth score, body mass index 10 years ago, tea drinking, history of oesophageal cancer among first-degree relatives, smoking status, alcohol drinking status, H. pylori sero-status, sum of missing and filled teeth and frequency of tooth brushing per day	P-B	7
	Fukuda et al. [33]	Japan	Case:282; control:767	GC	1989–1990	M/F	Low pepsinogen I/II ratio < 3.0	National Cancer Center Hospital records	Sex, age	H-B	7
	Gao et al. [34]	China	Case:315; control:1817	OJC	2010–2014	M/F	PGI/PGII ratio ≤ 4	Histopathological diagnosis from hospital	Age, sex, marital status, educational level, cigarette smoking, alcohol drinking, body mass index of 10 years ago, sum of missing and filled teeth, daily frequency of toothbrushing, family history of oesophageal cancer among first-degree relatives and Helicobacter pylori serostatus	P-B	8
	Iijima et al. [37]	Japan	Case:69; control:67	OC (OSCC)	2004–2006	M/F	Serum pepsinogen I < 25 ng/mL;	Histological diagnosis	Age, sex, BMI, smoking status, alcohol	H-B	7
			Case:71; control:70				Histology: Fundic atrophy, Fundic				8
			Case:73; control:73				Histology: intestinal metaplasia,				8
	Nasrollahzadeh et al. [42]	Iran	Case:293; control:524	OC (OSCC)	2003–2007	M/F	PGI < 55 μg/dL and PGII < 11.8 μg/dL	Pathologic diagnosis (ICD-O code C160-169)	Age, sex, Place of residence, ethnicity, alcohol consumption, tobacco or opium use, education level, and vegetable/fruit consumption	P-B	8
	Venerito et al. [45]	Germany	Case:75; control:75	OC (OSCC)	1999–2010	M/F	PG I level of ≤ 70 ng/mL and PG I/II ratios of ≤ 3.0;	Histological diagnosis from the hospital	Sex, age	H-B	8
			case:75; control:75				Histology: Fundic atrophy, Fundic				7
			case:75; control:75				Histology: intestinal metaplasia,				7
	Ye et al. [48]	Sweden	case:67; control:449	OC (OAC)	1995–1997	M/F	Pepsinogen I level ≤ 28 μg/L	A comprehensive organization and six regional tumor registries	Age, sex, years of education, body mass index, smoking status and level of consumption of fruits and vegetables	P-B	8
			Case:75; control:449	OC (OSCC)			Pepsinogen I level ≤ 28 μg/L				8
			Case:103; control:449	OJC(CC)			Pepsinogen I level ≤ 28 μg/L				8
Nested case–control	Aromaa et al. [29]	Finland	Case:84; control:146	GC	1961–1980 (follow-up mean:9.5)	M/F	Pepsinogen I < 49 μg/liter	Cancer registry	Age, sex, and municipality	P-B	9
	Cook et al. [30]	Finland	Case:79; control:94	OC (OSCC)	1985–2005 (diagnostic time median:9.94)	M	PGI/PGII ≤ 4	ATBC study (ICD-9 code 150, ICD-O-2 codes 8050–8078)	Age, date of blood draw, smoking, cigarettes per day, alcohol, education, BMI, fruit intake, and vegetable intake	P-B	9
	Hansen et al. [35]	Norway	Case:129; control:376	GC	1973–1986 (median follow-up time to diagnosis of cancer was 11.9)	M/F	Serum pepsinogen I:II < 2.5	Cancer registry data (ICD-O-2)	Gender, date of birth, date of serum sampling and serum source	P-B	8
			Case:44; control 132	OJC(CC)							8
	In et al. [39]	The United States	Case:70; control:141	GC	1993–2008 (GC diagnosis median was 6.7)	M/F	Pepsinogen I ≤ 70 μg/L and pepsinogen I/II ratio ≤ 3.0	Study update forms and medical record (ICD-O-2 codes C160-C169)	HP, Family hx of GC, education, smoking, BMI	P-B	8
			case:35; control:72	OJC(CC)			Pepsinogen I ≤ 70 μg/L and pepsinogen I/II ratio ≤ 3.0				8
	Sasazuki et al. [43]	Japan	Case:511; control:511	GC	1990–2004 (–)	M/F	Pepsinogen I levels ≤ 70 ng/mL and pepsinogen I/II ratio ≤ 3.0	Cancer registry for the JPHC Study (ICD-O code C160-169)	Age, gender, resident area, blood donation date, fasting timesat blood donation, smoking status, consumption of fish gut, green_yellow vegetables, other vegetables, fruit, green tea, body mass index, and family history of gastric cancer	P-B	9
	Watanabe et al. [47]	Japan	Case:45; control:225	GC	1987–1995 (diagnosis of gastric cancer mean: 3.2)	M/F	PG I level of ≤ 70 ng/mL and PG I/II ratios of ≤ 3.0	Gastric Cancer Registry System	Sex, age, and address	P-B	9
Cohort	De Vries et al. [31]	The Netherlands	126 Incident cases from 97,728 CAG; no CAG: Dutch general population	OC (OSCC)	1991–2006	M/F	Histology: mucosal atrophy, intestinal metaplasia, dysplasia	Histo- and cytopathology reports are collected in a national archive	Age categories, gender and calendar year	P-B	8
	Holleczek et al. [36]	Germany	CAG:281; no CAG:8805; (incident cases: CAG:5; no CAG:32)	GC	2000–2002 (follow-up average:13.8)	M/F	Severe: PG I < 20 ng/ml and PG I/II ratio < 3	Records from the cancer registry, ICD-10: C16	Age, sex, tobacco smoking, alcohol consumption, education, BMI and consumption of fruit and vegetables per day	P-B	9
	Ikeda et al. [38]	Japan	CAG:714; control:606 (incident cases: CAG:70; control:5)	GC	1988–2008	M/F	sPG I levels of ≤ 70 ng/mL and PG I/II ratios of ≤ 3.0	The guidelines of the Japan Gastric Cancer Association	Age, sex, body mass index, total cholesterol, hemoglobin A1c, smoking habits, and daily total energy and salt intake	P-B	8
	Inoue et al. [40]	Japan	CAG:8039; no CAG:11,067; (incident cases: CAG:431; no CAG:164)	GC	1993–2013 (follow-up average:18)	M/F	PG I ≤ 70 ng/mL and PG I/II ≤ 3.0	Hospital and cancer registry records (ICD-O-3;C16)	Sex, age at baseline, study area, smoking status, family history of gastric cancer and consumption of highly salted food	P-B	9
	Inoue et al. [41]	Japan	CAG:976; no CAG:4397; (incident cases: CAG:108; no CAG:9)	GC	1985–1999 (follow-up average:10)	M/F	Histology: mucosal atrophy	Records from hospitals and cancer registries	Age, gender and family history of gastric cancer	P-B	8
	Song et al. [44]	Sweden	CAG:14,285; normal:81,174 (incident cases: CAG:12; normal control:24)	OJC(CC)	1979–2011 (follow-up average:10)	M/F	Histology: mucosal atrophy, Intestinal metaplasia	Cancer register (ICD-7 code 151)	Sex	P-B	7
			CAG:11,530; normal:81,174 (incident cases: CAG:12; normal control:24)	OJC(CC)			Histology: Intestinal metaplasia				7
			CAG:14,285; normal:81,174 (incident cases: CAG:104; normal:97)	GC			Histology: mucosal atrophy,				7
			CAG:11,530; normal:81,174 (incident cases: CAG:76; normal control:24)	GC			Histology: intestinal metaplasia				7
	Vohlonen et al. [46]	Finland	CAG:606; healthy control:5232 (incident cases: CAG:2; healthy control:2)	OJC(CC)	1994–2013 (follow-up:15)	M	SPGI < 25 μg/L	Cancer registry	Sex	P-B	8
			Case:606; healthy control:5232 (incident cases: CAG:6; healthy control:5)	GC			SPGI < 25 μg/L				8

*OC* oesophageal cancer, *OSCC* oesophageal squamous cell carcinoma, *OAC* oesophageal adenocarcinoma, *GC* gastric cancer, *CC* cardia cancer, *OJC* oesophagogastric junction cancer, *FINBAR* factors influencing the barrett’s adenocarcinoma relationship, *ATBC* alpha-tocopherol beta-carotene cancer prevention, *JPHC* japan public health center, *H-B* hospital based, *P-B* population based, *C-B* clinic based

### Risk of gastric cancer

**Fig. 2:**
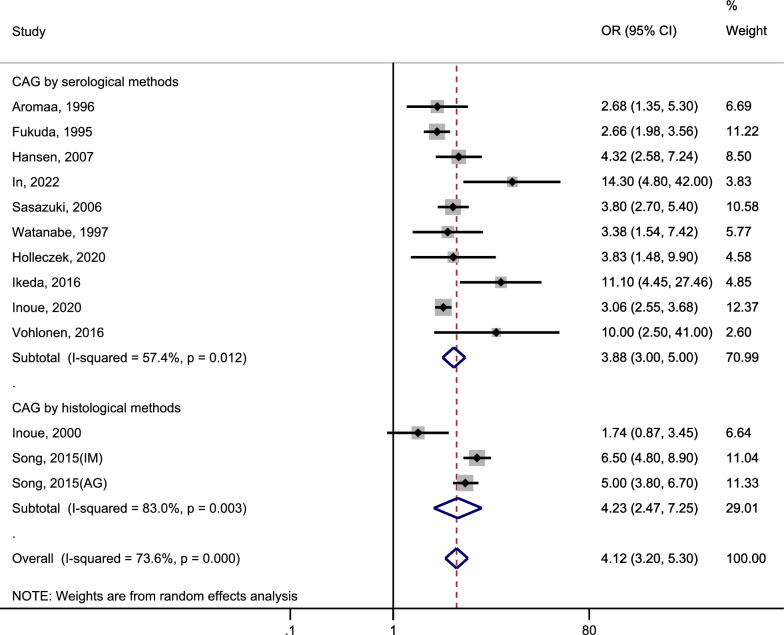
Forest plot to assess the relationship between CAG and gastric cancer. *CAG* chronic atrophic gastritis, *OR* odds ratio, *CI* confidence interval, *IM* intestinal metaplasia, *AG* atrophic gastritis

Thirteen studies were included to assess the relationship between CAG and the incidence of GC. The heterogeneity test (I^2^ = 73.6%, P < 0.1) indicated significant heterogeneity in this study. The pooled results were shown in Fig. 2 (Page 39): CAG was associated with a 4.12-fold increase in the risk of GC (pooled random effect OR = 4.12, 95%CI 3.20 ~ 5.30); The risk of incident GC, when diagnosed through histological methods for CAG, was higher (OR = 4.23, 95% CI 2.47–7.25) compared to the risk associated with diagnosing CAG through serological methods (OR = 3.88, 95% CI 3.00–5.00).

Significant heterogeneity was observed in this study. In order to delve the origins of heterogeneity, we conducted subgroup analyses based on the diagnosis method of CAG, study type, participant source, region, year of publication, and NOS score. The relevant results are presented in Table 2 (Page 36). There was no significant heterogeneity in nested case–control studies, European studies, or studies published between 1995 and 2010. However, significant heterogeneity was detected in all other subgroup analyses. In all subgroups, patients with CAG had a significantly increased risk of incident GC. Particularly, studies conducted in the United States showed the highest relative risk of GC incidence among patients with CAG (OR = 14.30, 95% CI 4.83–42.30). This study was both a case–control and a hospital-based study, and it showed that patients with CAG had a relatively low risk of developing GC (OR = 2.66, 95% CI 1.98–3.57). In other subgroup analyses, the risk of incident GC was very similar to the overall pooled risk.

**Table 2:** Association of CAG with gastric cancer risk: a subgroup analysis

Characteristic	Stratified analysis	No. of studies	Pooled OR (95%CI)^a^	Heterogeneity	
				I^2^ (%)	*p* value^b^
	All studies	13	4.12 (3.20, 5.30)	73.6	< 0.001
Diagnosis					
	By serological method	10	3.88 (3.00, 5.00)	57.4	0.012
	By histological method	3	4.23 (2.47, 7.25)	83	0.003
Study type					
	Nested case–control	5	4.08 (2.85, 5.86)	42.5	0.138
	Case–control study	1	2.66 (1.98, 3.57)	–	–
	Cohort study	7	4.54 (3.06, 6.73)	81.4	< 0.001
Participant source					
	Population based	12	4.36 (3.33, 5.70)	71.9	< 0.001
	Hospital based	1	2.66 (1.98, 3.57)	–	–
Region					
	Japan	6	3.22 (2.46, 4.23)	60.6	0.026
	Europe	6	4.96 (3.85, 6.38)	32.6	0.192
	The US	1	14.30 (4.83, 42.30)	–	–
Year of publication					
	1995–2010	10	3.08 (2.44, 3.88)	27.3	0.230
	2011–2023	3	5.74 (3.82, 8.62)	80.9	< 0.001
NOS score					
	NOS score ≥ 8	10	3.94 (2.94, 5.29)	59.4	0.008
	NOS score < 8	3	4.42 (2.64, 7.39)	89.0	< 0.001

*CAG* chronic atrophic gastritis, *OR* odds ratio, *CI* confidence interval, *The US:* the United States, *NOS* newcastle–ottawa scale

^a^A random-effect model was adopted

^b^*p* value from Q-test

### Risk of oesophageal cancer

**Fig. 3:**
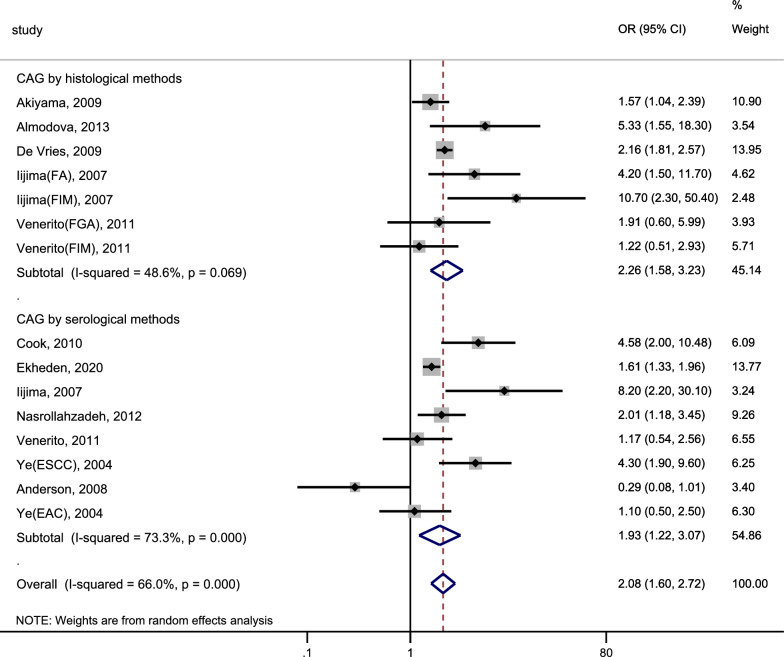
Forest plot to assess the relationship between CAG and osophageal cancer. *CAG* chronic atrophic gastritis, *OR* odds ratio, *CI* confidence interval, *FA* fundic atrophy, *FIM* fundic intestinal metaplasia, *FGA* fundic gastric atrophy, *OSCC* oesophageal squamous cell carcinoma, *OAC* oesophageal adenocarcinoma

**Fig. 4:**
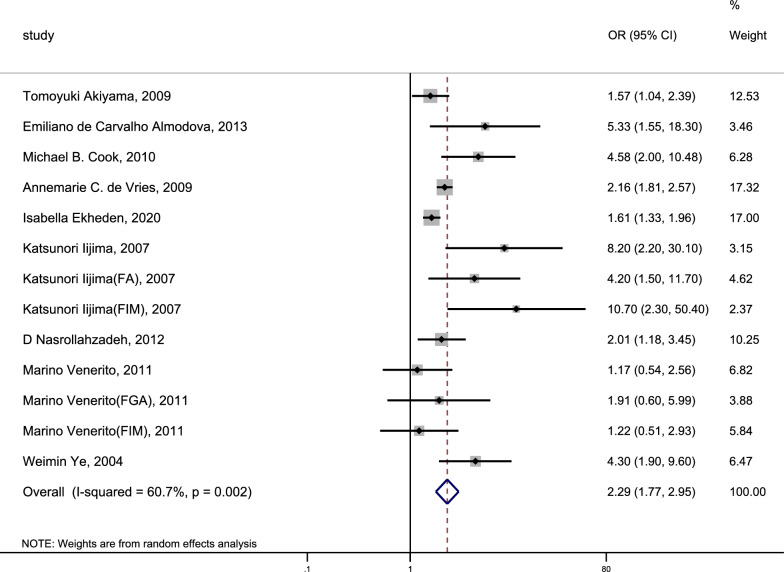
Forest plot to assess the relationship between CAG and oesophageal squamous cell carcinoma. *CAG* chronic atrophic gastritis, *OR* odds ratio, *CI* confidence interval, *FA* fundic atrophy, *FIM* fundic intestinal metaplasia, *FGA* fundic gastric atrophy

**Fig. 5:**
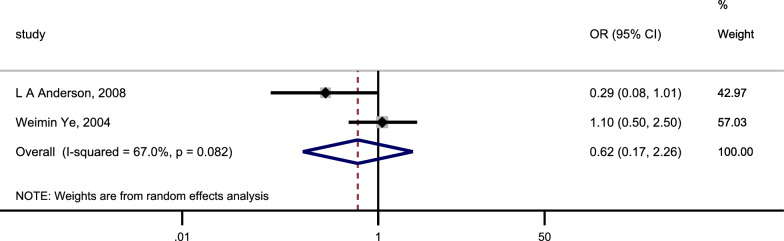
Forest plot to assess the relationship between CAG and oesophageal adenocarcinoma. *CAG* chronic atrophic gastritis, *OR* odds ratio, *CI* confidence interval

We included 15 studies that explored the association between CAG and the incidence of OC. The heterogeneity analysis indicated significant heterogeneity within this research (I^2^ = 66.0%, P < 0.1). The pooled results were shown in Fig. 3 (Page 39).: CAG was associated with a 2.08-fold increase in the risk of incident OC (pooled random-effect OR = 2.08, 95% CI 1.60–2.72). The risk of incident OC was markedly higher with the diagnosis of CAG through histologic methods (OR = 2.26, 95% CI 1.58–3.23) compared to the risk associated with diagnosing CAG through serologic methods (OR = 1.93, 95%CI 1.22–3.07). Meanwhile, we assessed the relationship between CAG and the risk of incident OSCC and OAC (Figs. 4, 5; Page 40). Our findings indicated that CAG was linked to a 2.29-fold increase in the risk of incident OSCC (pooled random-effects OR = 2.29, 95% CI 1.77–2.95, I^2^ = 60.7%, P = 0.002). Nevertheless, there was no significant association between CAG and the risk of incident OAC (pooled random effect OR = 0.62, 95% CI 0.17–2.26, I^2^ = 67.0%, P = 0.082).

In order to explore the sources of heterogeneity, we performed subgroup analyses based on the diagnosis method of CAG, study type, participant source, region, year of publication, and NOS score. As shown in Table 3 (Pages 37, 38), there was no significant heterogeneity in studies where CAG diagnosis was based on histologic methods and those published from 2011 to 2023. However, significant heterogeneity was observed in all other subgroup analyses. Additionally, studies with an NOS score < 8 did not reveal a significant association between CAG and the risk of OC, whereas all other subgroup analyses indicated a significant correlation. In the nested case–control study (OR = 4.58, 95%CI 2.00–10.48) and the study conducted in the Americas (OR 5.33, 95%CL 1.55–18.30), patients with CAG had a relatively higher risk of incident OC. In studies published from 2011 to 2023, the risk of OC in patients with CAG was relatively lower (OR = 1.66, 95%CI 1.35–2.04). In all other subgroup analyses, the risk of incident cancer was similar to the overall pooled risk.

**Table 3:** Association of CAG with oesophageal cancer risk: a subgroup analysis

Characteristic	Stratified analysis	No. of studies	Pooled OR (95%CI)^a^	Heterogeneity	
				I^2^ (%)	*p* value^b^
	All studies	15	2.08 (1.60, 2.72)	66.0	< 0.001
Diagnosis					
	By serological method	8	1.93 (1.22, 3.07)	73.3	< 0.001
	By histological method	7	2.26 (1.58, 3.23)	48.6	0.069
Study type					
	nested case–control	1	4.58 (2.00, 10.48)	–	–
	case–control study	13	1.99 (1.42, 2.78)	63.8	0.001
	cohort study	1	2.16 (1.81, 2.57)	–	–
Participant Source					
	Population based	7	1.92 (1.38, 2.68)	74.3	0.001
	Hospital based	8	2.57 (1.51, 4.36)	60.6	0.013
Region					
	Asia	6	2.36 (1.56, 3.59)	65.9	0.012
	Europe	8	1.73 (1.10, 2.71)	68.1	0.003
	America	1	5.33 (1.55, 18.31)	–	–
Year of publication					
	1995–2010	9	2.48 (1.58, 3.88)	73.6	< 0.001
	2011–2023	6	1.66 (1.35, 2.04)	6.0	0.378
NOS score					
	NOS score ≥ 8	9	2.26 (1.65, 3.09)	58.0	0.015
	NOS score < 8	6	1.82 (0.93, 3.55)	70.6	0.004

*CAG*, chronic atrophic gastritis, *OR* odds ratio, *CI* confidence interval, *NOS* newcastle–ottawa scale

^a^A random-effect model was adopted

^b^*p* value from Q-test

### Risk of oesophagogastric junction cancer

**Fig. 6:**
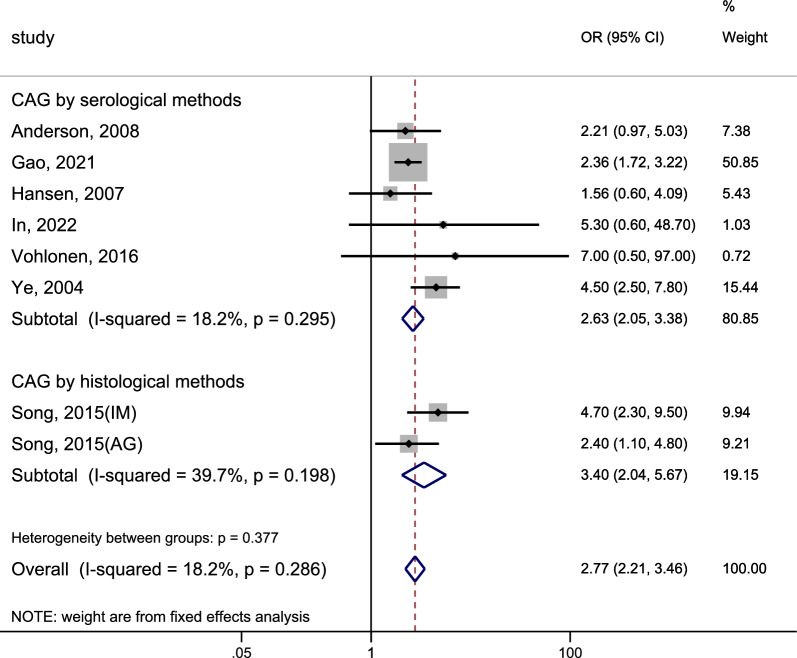
Forest plot to assess the relationship between CAG and oesophagogastric junction cancer. *CAG* chronic atrophic gastritis, *OR* odds ratio, *CI* confidence interval

We included 8 studies to examine the association between CAG and the risk of incident OJC. The heterogeneity test indicated a low level of heterogeneity in this study (I^2^ = 18.2%, P = 0.286 > 0.1). The pooled results were displayed in Fig. 6 (Page 40): CAG was associated with a 2.77-fold increased risk of OJC (pooled fixed effect OR = 2.77, 95% CI 2.21–3.46). The risk of OJC was significantly higher when CAG was diagnosed through histological methods (OR = 3.40, 95%CI 2.04–5.67) compared to serological methods (OR = 2.63, 95%CI 2.05–3.38), and neither of these groups of studies displayed significant heterogeneity.

### Sensitivity analyses and publication bias

**Fig. 7:**
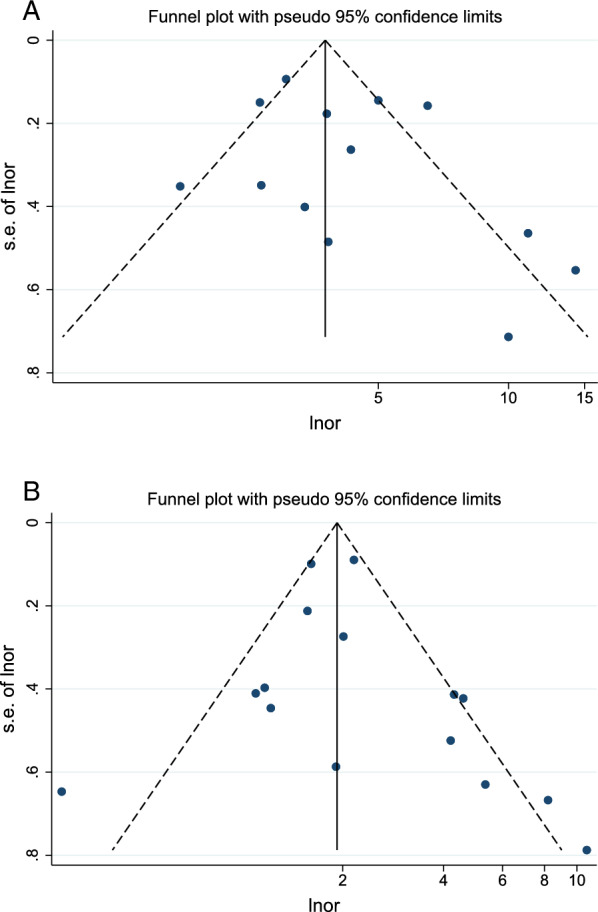
Publication bias. **A** Funnel plot of studies assessing the relationship between CAG and risk of gastric cancer. **B** Funnel plot of studies assessing the relationship between CAG and risk of oesophageal cancer

**Fig. 8:**
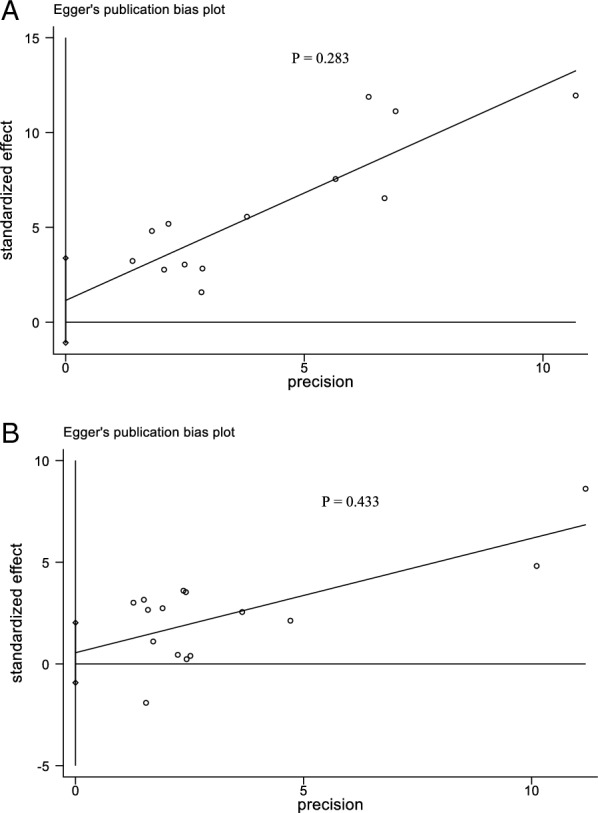
Publication bias. **A** Egger’ s test evaluating for the relationship between CAG and risk of gastric cancer. **B** Egger’ s test evaluating for the relationship between CAG and risk of oesophageal cancer

For GC, OC and OJC, we performed sensitivity analyses using a study-by-study exclusion approach, and our findings demonstrated the stability and reliability of the pooled results (Additional file 2: Fig. S1a–c). To evaluate publication bias in GC and OC, we used funnel plots and Egger's tests. Visual inspection of the funnel plots (Fig. 7) (Page 41) showed that the distributions were generally symmetrical, indicating that there was no significant publication bias. The results of the Egger's test (Fig. 8) (Pages 41, 42) indicated that, in the analysis of GC (P = 0.283) and OC (P = 0.433), no significant publication bias was observed in the studies.

## Discussion

In this meta-analysis, we included a total of 23 studies involving 5858 patients diagnosed with upper gastrointestinal cancers. Our objective was to analyze the connection between CAG and the incidence of upper gastrointestinal cancers. The results clearly pointed to a significant 4.12-fold elevation in the risk of GC, a 2.77-fold increase in the risk of OJC, and a 2.08-fold rise in the risk of OC among patients with CAG. Furthermore, our findings indicated a 2.29-fold increased risk of OSCC in CAG patients. However, no significant association was detected with the risk of OAC. Intriguingly, when considering the same upper gastrointestinal cancer, the risk of developing cancer was higher with CAG diagnosed through histologic methods rather than serologic methods.

This study represents the first comprehensive assessment of the association between CAG and the incidence of upper gastrointestinal cancers. Our findings reveal a substantial increase in the risk of upper gastrointestinal cancers linked to CAG, which is both consistent and inconsistent with previously published meta-analyses in different regards. Previously, gastric atrophy has been correlated with a 2.89-fold elevated risk of Cardia Cancer when diagnosed through serologic methods [12]. In contrast, our current study explores the risk of CAG and the incidence of OJC, employing both histologic and serologic diagnostic methods. Additionally, Gastric Atrophy has previously exhibited associations with the risk of OSCC and OAC, with a 1.94-fold heightened risk of OSCC incidence but a reduced risk of OAC development [12]. In this study, we have not only reported the link between CAG and an elevated risk for developing OSCC and OAC but have also indicated its relevance to the risk of developing EC. Worth noting is that patients with Intestinal Metaplasia (IM) have been previously reported to exhibit a significant 3.58-fold increase in the risk of GC, particularly when IM develops in the gastric body or presents as incomplete IM [42]; A systematic review and meta-analysis conducted by Sui [43] indicated that there was a significant 2.91-fold increase in the risk of GC associated with gastric atrophy; These results correspond with the trends observed in our own study. Furthermore, Sui's study further reported that the risk of developing GC was higher with CAG diagnosed through serum pepsinogen levels rather than endoscopy [43]. Interestingly, this result contradicts the findings of our study.

We hypothesize several potential mechanisms underlying the association between CAG and upper gastrointestinal cancers. First and foremost, CAG is predominantly attributed to H. pylori infection. H. pylori can generate a multitude of virulence factors that target gastric mucosal tissues, disrupting intracellular signalling pathways and lowering the threshold for tumour transformation. Notably, the primary virulence factors of H. pylori include cytotoxin-associated gene A (CagA) and its pathogenicity island (Cag PAI), as well as vacuolating cytotoxin A (VacA). The Wnt signalling pathway, known for its role in cancer development, is implicated in GC through the upregulation of Wnt10A in gastric mucosa-associated cells, further activating the Wnt-β-catenin-Tcf signalling pathway, significantly contributing to GC development [44]. Similarly, the upregulation of Wnt10A may also be a factor in OC development [45], as subsequent studies have indicated its enhancement of invasion and migration in OSCC [46]. Secondly, CAG induced by H. pylori infection is characterized by reduced or complete abstention of gastric acid secretion, leading to the creation of a hypochlorhydric microenvironment. Such a microenvironment fosters the proliferation of oncogenic microorganisms within the stomach and augments the production of N-nitroso compounds, which significantly increases the risk of GC and OSCC [47–49]. The incidence risk of OAC is positively linked to gastroesophageal reflux symptoms [50] but negatively associated with H. pylori infection [41]. Consequently, CAG triggered by H. pylori infection would seem to either reduce the occurrence of OAC or have no discernible impact. The risk of OJC may exhibit two potential scenarios: some cases, similar to OAC, OJC could have a negative correlation or no association with CAG. meanwhile, others, resembling GC, may show a positive correlation with CAG. Lastly, chronic inflammation is one of the potential mechanisms contributing to cancer development, and this applies equally to upper gastrointestinal cancers. When tissue damage occurs, inflammatory cells gather and release inflammatory cytokines, thereby promoting the generation of reactive oxygen species (ROS). These ROS induce cellular proliferation, causing oxidative damage to DNA and, in turn, amplifying the risk of cancer development [4]. CAG remains in a chronic inflammatory state, particularly following H. pylori infection, which triggers the upregulation of multiple pro-inflammatory factors, including interleukin-8 (IL-8), nuclear factor-κB (NF-κB), tumour necrosis factor-α (TNF-α), and interleukin-6 (IL-6) and others. The upregulation of IL-8 and activation of NF-κB in gastric epithelial cells play pivotal roles in the mechanisms underlying chronic inflammation and the development of GC [51]. Additionally, NF-κB is closely associated with metastasis, inflammation, and poor prognosis in OC patients [52].

This meta-analysis reveals a certain degree of heterogeneity. To ensure the robustness of our findings, we conducted a sensitivity analysis, which confirmed the stability of the pooled results. In our assessment of GC and OC studies, both funnel plots and Egger's tests were employed, and the results consistently showed no clear evidence of publication bias.

In the subgroup analyses, notable findings emerged. In the GC study, all subgroup analyses consistently indicated a significant increase in GC risk among patients with CAG. It is worth highlighting that the studies conducted in the United States (US) reported the highest incidence of GC, even though GC is relatively uncommon in the US. This discovery emphasizes the potential relevance of serum pepsin as a predictive marker for GC in the US [32]. Among Asian research, Japan remains the sole contributor to relevant studies, emphasizing the necessity for broader participation from other Asian regions in future observational research. Notably, there was a study that served as both a case–control study and hospital-based research, revealing a comparatively lower risk level, possibly associated with the control group's population selection process. In the field of OC research, studies with NOS scores below 8 did not reveal any significant risk association. Nevertheless, all other subgroup analyses consistently pointed to a marked increase in OC incidence risk associated with CAG. Studies conducted in Latin America indicated the highest risk of OC, whereas research published between 2011 and 2023 showed a comparatively lower OC risk. Hospital-based studies showed a comparatively higher OC risk than population-based studies, possibly due to the inclusion of more severe cases from hospital settings.

Another crucial aspect of our study is the exploration of the association between CAG diagnosed through two different diagnostic methods and the risk of upper gastrointestinal cancers. In studies assessing GC risk in relation to CAG, histological confirmation of CAG was linked to a 35% increased risk of GC compared to serological diagnosis. Similarly, regarding the relationship between CAG and OC risk, histologic diagnosis of CAG was connected to a 33% higher risk of OC compared to serologic diagnosis. Moreover, in the investigation of the risk of OJC associated with CAG, histological confirmation of CAG was associated with a 77% elevated risk of OJC compared to serological diagnosis. In general, for GC, OC and OJC, the risk of cancer development was linked to a 33%-77% higher when CAG is diagnosed histologically compared to serologically. It's important to note that the guidelines for managing precancerous gastric epithelial lesions and other lesions recommend serum pepsinogen level as the best noninvasive test for detecting atrophic gastritis. However, in cases of low serum pepsinogen levels, reliance on gastroscopy is necessary [53]. The accuracy of endoscopic biopsy results can be influenced by a range of factors, including the quality of biopsy samples, specimen handling, and the expertise of pathologists [54]. Furthermore, the use of different analytical methods and threshold values with serum pepsinogen diagnosis can result in varying levels of specificity and sensitivity [55]. Therefore, further research and investigation are essential to comprehensively assess the risk associated with CAG diagnosis for upper gastrointestinal cancers using these two methods.

Our meta-analysis has also additional important strengths. As previously mentioned, our study aims to provide the most comprehensive evaluation of the connection between CAG and the risk of upper gastrointestinal cancers to date. We have included data from Asia, Europe, and the Americas, ensuring that the selected studies adhere to a high standard of quality.

However, there are certain limitations to our study. Firstly, the pooled results are constrained by the scarcity of studies focusing on the association between CAG and the risk of incident OAC. Future large-scale observational studies are imperative to delve deeper into the relationship between CAG and the incidence of OAC. Furthermore, the studies we have incorporated into our analysis display differences in adjusted factors and involve differing study designs, potentially introducing additional bias into the pooled findings. The data in our study was primarily sourced from medical records and cancer registries, which might introduce a degree of selection bias into the dataset. Finally, the primary diagnostic modalities for CAG include endoscopic histology and serum pepsinogen levels. While these two methods are widely used in current medical practice, they still exhibit certain limitations. The former can be influenced by factors such as the endoscopist's skill, specimen handling protocols, and diagnostic interpretation by the pathologist, whereas the results of the latter may fluctuate based on specimen analysis techniques and the selection of critical values.

## Conclusion

This meta-analysis showed a two- to fourfold increased risk of GC, EC and EJC in patients with CAG. Importantly, for the same upper gastrointestinal cancer, the risk of incident cancer was higher when CAG was diagnosed through histological methods compared to serological methods. Further rigorous study designs are required to explore the impact of CAG diagnosed through both diagnostic methods on the risk of upper gastrointestinal cancers.

### Supplementary Information

**Additional file 1: File 1** Search strategy.

**Additional file 2: Fig. S1.** Sensitivity analysis was performed for gastric cancer (GC), oesophageal cancer (OC), and oesophagogastric junction cancer (OJC) using a study-by-study exclusion approach.

## Data Availability

All data generated or analyzed during this study are included in this published article [and its Additional files].

## Abbreviations

CAG: Chronic atrophic gastritis
OR: Odds ratio
CI: Confidence interval
GC: Gastric cancer
OJC: Oesophagogastric junction cancer
OC: Oesophageal cancer
OAC: Oesophageal adenocarcinoma
OSCC: Oesophageal squamous cell carcinoma
PRISMA: Preferred Reporting Items for Systematic Reviews and Meta-Analyses
NOS: Newcastle–Ottawa Scale score
HR: Hazard ratio
RR: Relative risk
IM: Intestinal metaplasia
AG: Atrophic gastritis
FA: Fundic atrophy
FIM: Fundic intestinal metaplasia
FGA: Fundic gastric atrophy
IL-8: Interleukin-8
NF-κB: Nuclear factor-κB
TNF-α: Tumour necrosis factor-α
IL-6: Interleukin-6
The US: The United States
