# Supplementary material for: Chronic atrophic gastritis and risk of incident upper gastrointestinal cancers: a systematic review and meta-analysis
Source: J Transl Med. 2024 May 6;22:429. doi: 10.1186/s12967-023-04736-w (PMC11075312; doi:10.1186/s12967-023-04736-w)
Supplement: Supplementary file 1 — Additional file 1: File 1 Search strategy. [file 12967_2023_4736_MOESM1_ESM.pdf]

Supplementary File 1 Search strategy
Date 2023.8.10
Databases searched: PubMed, EMBASE, Web of Science,

PubMed:
Search: ((("Gastritis, Atrophic"[Mesh]) OR ((((atrophic gastritides[Title/Abstract]) OR (atrophic gastritis[Title/Abstract])) OR (gastritides, atrophic[Title/Abstract])) OR (Chronic atrophic gastritis[Title/Abstract]))) AND ((((((stomach[Title/Abstract]) OR (gastric[Title/Abstract])) OR (cardia[Title/Abstract])) OR (oesophag*[Title/Abstract])) OR (esophag*[Title/Abstract])) OR (("Esophagogastric Junction"[Mesh]) OR (((((junction, esophagogastric[Title/Abstract]) OR (gastroesophageal junction[Title/Abstract])) OR (gastroesophageal junctions[Title/Abstract])) OR (junction, gastroesophageal[Title/Abstract])) OR (junctions, gastroesophageal[Title/Abstract]))))) AND (("Neoplasms"[Mesh]) OR ((((((((((((((((((tumor[Title/Abstract]) OR (tumors[Title/Abstract])) OR (neoplasm[Title/Abstract])) OR (neoplasia[Title/Abstract])) OR (neoplasias[Title/Abstract])) OR (cancer[Title/Abstract])) OR (cancers[Title/Abstract])) OR (malignant neoplasm[Title/Abstract])) OR (malignancy[Title/Abstract])) OR (malignancies[Title/Abstract])) OR (malignant neoplasms[Title/Abstract])) OR (neoplasm, malignant[Title/Abstract])) OR (neoplasms, malignant[Title/Abstract])) OR (benign neoplasms[Title/Abstract])) OR (benign neoplasm[Title/Abstract])) OR (neoplasms, benign[Title/Abstract])) OR (neoplasm, benign[Title/Abstract])) OR (carcinoma[Title/Abstract])) OR (adenocarcinoma[Title/Abstract])))

EMBASE:
#1 'atrophic gastritis'/exp
#2 'atrophic gastritides':ab,ti OR 'atrophic gastritis':ab,ti OR 'gastritides, atrophic':ab,ti OR 'chronic atrophic gastritis':ab,ti
#3 #1 OR #2
#4 'stomach':ab,ti OR 'gastric':ab,ti OR 'cardia':ab,ti OR 'oesophag*':ab,ti OR 'esophag*':ab,ti
#5 'gastroesophageal junction'/exp
#6 'junction, esophagogastric':ab,ti OR 'gastroesophageal junction':ab,ti OR 'gastroesophageal junctions':ab,ti OR 'junction, gastroesophageal':ab,ti OR 'junctions, gastroesophageal':ab,ti
#7 #5 OR #6
#8 #4 OR #7
#9 'malignant neoplasm'/exp
#10'tumor':ab,ti OR 'tumors':ab,ti OR 'neoplasm':ab,ti OR 'neoplasia':ab,ti OR 'neoplasias':ab,ti OR 'cancer':ab,ti OR 'cancers':ab,ti OR 'malignant neoplasm':ab,ti OR 'malignancy':ab,ti OR 'malignancies':ab,ti OR 'malignant neoplasms':ab,ti OR

'neoplasm, malignant':ab,ti OR 'neoplasms, malignant':ab,ti OR 'benign neoplasms':ab,ti OR 'benign neoplasm':ab,ti OR 'neoplasms, benign':ab,ti OR 'neoplasm, benign':ab,ti OR 'carcinoma':ab,ti OR 'junction, esophagogastric':ab,ti OR 'gastroesophageal junction':ab,ti OR 'gastroesophageal junctions':ab,ti OR 'junction, gastroesophageal':ab,ti OR 'junctions, gastroesophageal':ab,ti
#11 #9 OR #10
#12 #3 AND #8 AND #11

Web of Science:
#1 ((((TS=(gastritis, atrophic)) OR TS=(atrophic gastritides)) OR TS=(atrophic gastritis)) OR TS=(gastritides, atrophic)) OR TS=(Chronic atrophic gastritis) and Preprint Citation Index
#2 TS=(stomach OR gastric OR cardia OR oesophag* OR esophag*) and Preprint Citation Index
#3 TS=(esophagogastric junction OR junction, esophagogastric OR gastroesophageal junction OR gastroesophageal junctions OR junction, gastroesophageal OR junctions, gastroesophageal) and Preprint Citation Index
#4 #2 OR #3 and Preprint Citation Index
#5 TS=(neoplasms OR tumor OR tumors OR neoplasm OR neoplasia OR neoplasias OR cancer OR cancers OR malignant neoplasm OR malignancy OR malignancies OR malignant neoplasms OR neoplasm, malignant OR neoplasms, malignant OR benign neoplasms OR benign neoplasm OR neoplasms, benign OR neoplasm, benign OR carcinoma OR adenocarcinoma) and Preprint Citation Index
#6 #1 AND #4 AND #5 and Preprint Citation Index
