# Supplementary material for: Chronic atrophic gastritis and risk of incident upper gastrointestinal cancers: a systematic review and meta-analysis
Source: J Transl Med. 2024 May 6;22:429. doi: 10.1186/s12967-023-04736-w (PMC11075312; doi:10.1186/s12967-023-04736-w)
Supplement: Supplementary file 2 — Additional file 2: Fig. S1. Sensitivity analysis was performed for gastric cancer (GC), oesophageal cancer (OC), and oesophagogastric junction cancer (OJC) using a study-by-study exclusion approach. [file 12967_2023_4736_MOESM2_ESM.doc]

Sensitivity analysis was performed for gastric cancer (GC), oesophageal cancer (OC), and oesophagogastric junction cancer (OJC) using a study-by-study exclusion approach, as illustrated in Additional file 1: Fig. S1a-c.


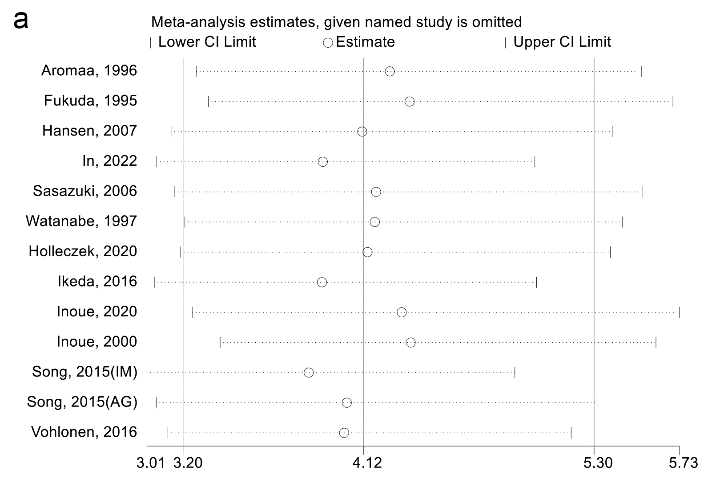


Figure S1a: Sensitivity analysis of chronic atrophic gastritis(CAG) and risk of gastric cancer(GC) development.


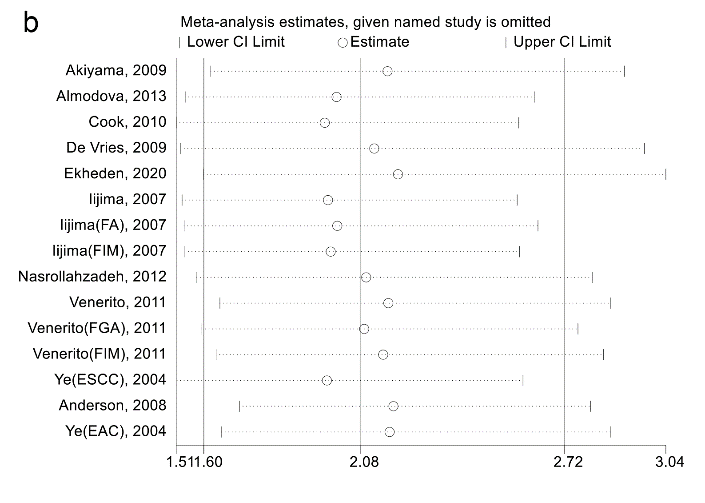


Figure S1b: Sensitivity analysis of chronic atrophic gastritis(CAG) and risk of oesophageal cancer (OC) development.


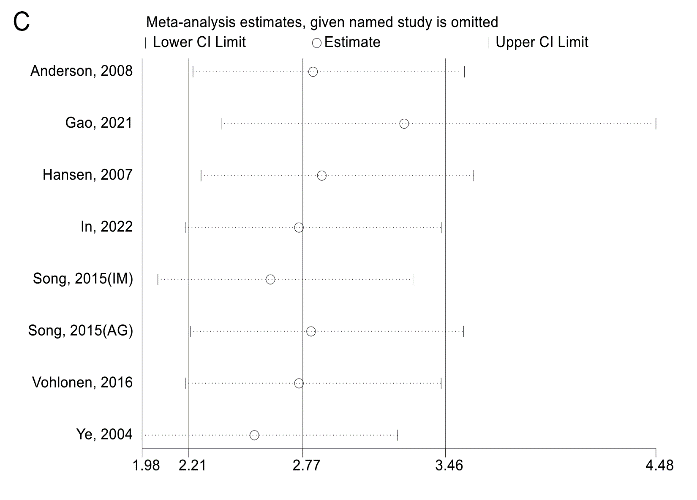


Figure S1c: Sensitivity analysis of chronic atrophic gastritis(CAG) and risk of oesophagogastric junction cancer (OJC) development.
